# The Applicability of Rhythm-Motor Tasks to a New Dual Task Paradigm for Older Adults

**DOI:** 10.3389/fneur.2017.00671

**Published:** 2017-12-22

**Authors:** Soo Ji Kim, Sung-Rae Cho, Ga Eul Yoo

**Affiliations:** ^1^Music Therapy Education, Graduate School of Education, Ewha Womans University, Seoul, South Korea; ^2^Department and Research Institute of Rehabilitation Medicine, Yonsei University College of Medicine, Seoul, South Korea; ^3^Brain Korea 21 PLUS Project for Medical Science, Yonsei University College of Medicine, Seoul, South Korea; ^4^Rehabilitation Institute of Neuromuscular Disease, Yonsei University College of Medicine, Seoul, South Korea; ^5^Department of Music Therapy, Graduate School, Ewha Womans University, Seoul, South Korea

**Keywords:** dual task, gait control, rhythmic cueing, dual task interference, elderly

## Abstract

Given the interplay between cognitive and motor functions during walking, cognitive demands required during gait have been investigated with regard to dual task performance. Along with the needs to understand how the type of concurrent task while walking affects gait performance, there are calls for diversified dual tasks that can be applied to older adults with varying levels of cognitive decline. Therefore, this study aimed to examine how rhythm-motor tasks affect dual task performance and gait control, compared to a traditional cognitive-motor task. Also, it examined whether rhythm-motor tasks are correlated with traditional cognitive-motor task performance and cognitive measures. Eighteen older adults without cognitive impairment participated in this study. Each participant was instructed to walk at self-paced tempo without performing a concurrent task (single walking task) and walk while separately performing two types of concurrent tasks: rhythm-motor and cognitive-motor tasks. Rhythm-motor tasks included instrument playing (Walk_IP_), matching to rhythmic cueing (Walk_RC_), and instrument playing while matching to rhythmic cueing (Walk_IP+RC_). The cognitive-motor task involved counting forward by 3s (Walk_Count.f3_). In each condition, dual task costs (DTC), a measure for how dual tasks affect gait parameters, were measured in terms of walking speed and stride length. The ratio of stride length to walking speed, a measure for dynamic control of gait, was also examined. The results of this study demonstrated that the task type was found to significantly influence these measures. Rhythm-motor tasks were found to interfere with gait parameters to a lesser extent than the cognitive-motor task (Walk_Count.f3_). In terms of ratio measures, stride length remained at a similar level, walking speed greatly decreased in the Walk_Count.f3_ condition. Significant correlations between dual task-related measures during rhythm-motor and cognitive-motor tasks support the potential of applying rhythm-motor tasks to dual task methodology. This study presents how rhythm-motor tasks demand cognitive control at different levels than those engaged by cognitive-motor tasks. It also indicates how these new dual tasks can effectively mediate dual task performance indicative of fall risks, while requiring increased cognitive resources but facilitating gait control as a compensatory strategy to maintain gait stability.

## Introduction

Increased fall risks and fall-related serious injuries associated with aging are a public health concern (1, 2). Since many daily tasks are performed while people are standing or walking, increased risk of falling among the elderly leads to changes in their life style, including a tendency to become sedentary and avoid physical activity. Such changes often result in decreased quality of life (3). Furthermore, given that falls have been documented to be associated with a high prevalence of comorbidities or increased mortality, understanding risk factors for falls and strategies to intervene in age-related changes in gait is of great interest.

Factors for increased incidence of falls in older adults include decrements in gait function, such as decreased walking speed and shorter stride length (4–6). Increased variability in gait parameters such as unsteadiness in stride-to-stride timing is also a predictive factor for falls since it affects the ability to optimally adjust gait in response to changes in the environment (1). Also of interest is the relationship between spatiotemporal gait parameters (e.g., different combination of step frequency, walking speed, and stride length). Since gait control and stability are influenced by not a single parameter but a combination of gait parameters (7), the ratio of stride length to walking speed demonstrates how such a combination mediates gait differently. For healthy walkers without gait impairment, a shorter stride length and increased step frequency while maintaining walking speed reflects an adaptive strategy to maintain dynamic stability during walking (8, 9). Meanwhile, older adults with a history of falling tend to show decreases in walking speed as well as in step frequency and stride length.

Along with age-related changes in physical ability, cognitive control at a higher level is increasingly documented to affect walking (10, 11). While gait itself is regarded as an automatic motor function regulated primarily by subcortical processes, recent findings suggest that cortical areas are involved in dual task walking in parallel (11–13), which indicates that a higher level of cognitive control is involved while walking. As such, previous studies indicate that multiple cognitive processes, including attention, working memory, and executive function, mediate gait, especially when walking is required in a challenging environment with the presence of obstacles (12) or with the provision of concurrent tasks (14).

Investigation of dual task paradigms in walking suggests reduced cognitive capacity yields limited access to control cognitive loads during dual task walking (15, 16). Changes in gait parameters when walking with concurrent tasks, also measured as dual task costs (DTC), are indicative of the extent of how much dual task condition leads to deterioration of such task performance due to cognitive demands involved in gait (17). For example, when individuals walk and perform cognitive tasks simultaneously, additional attention is involved to achieve gait stability despite concurrent performance of gait and accordingly, gait speed is altered (18, 19). As such, involvement of competing demands for limited attentional or mental resources has been understood as a key factor in gait performance under dual task conditions (20, 21), yet the level of interference caused by different types of concurrent tasks is of great interest to researchers and practitioners who focus on dual task performance in relation to fall issues with older adults (22).

A diverse range of cognitive tasks in dual task walking studies exists, and recent findings indicate that the type and complexity of a cognitive task significantly influence motor outcomes in dual task walking (23). The tasks frequently cited in the literature are arithmetic (e.g., involving counting or operation of numbers), working memory or verbal fluency tasks associated with spoken verbal responses (11, 24). In terms of the type of cognitive tasks, no significant changes in walking were evidenced with verbal fluency tasks (25); however, walking while performing an arithmetic task yielded a significant decline in gait speed (22). Tasks involving internal interference, such as mental tracking and working memory, appear to interrupt gait more than tasks that involve external factors (24). In other words, gait function is decreased in the presence of an internal source of interference such as in the task to hold and keep track of information while performing ongoing mental processes.

Given the implications that the dual task paradigm may have not only for healthy older adults but also for the clinical population, including older adults with cognitive impairment and individuals with neurological disorders (24), there are increasing calls for diversified types of dual tasks. While verbal fluency tasks are one of the most commonly used and can be easily applied to older adults with varying levels of cognitive impairment, they do not involve substantial gait interference, leading to limited application to healthy populations who have relatively little decrements in gait function. For individuals with neurological disorders whose gait is affected, there is a need for interventions that target gait function as well in dual task paradigm (21, 24). These findings imply that dual task interference can be modified not only by altering the complexity of cognitive tasks, but also by regulating automated motor tasks (i.e., gait) to require increased use of attentional resources within the dual task paradigm. Moreover, the similarities and differences across the recruited resources can influence information processing (26). For example, the type and perceived complexity of the cognitive tasks in dual task walking can be controlled by the use of similar resources, such as shared sensory modalities. While research supports the use of cognitive concurrent tasks with a certain level of complexity, based on the evidence that complex tasks involving internal interfering factors affect gait disturbance more (24), research into the potential of tasks using external resources, which may be applied to older adults with limited internal resources or individuals with neurological disorders who need motor control as well as cognitive control, is inconclusive.

Therefore, this study proposed a new dual task paradigm incorporating a concurrent task that involves external auditory resources (i.e., rhythmic cueing) for motor control (i.e., rhythm-motor task). In the literature, walking with external auditory cueing was found to influence gait control (27) by establishing the feedback-feedforward loop that contributes to decreased attentional demands and increased continuous control of gait variability. When rhythmic auditory cueing was given during treadmill walking, it involved additional demands for balance and gait control, which was supported by a delay in reaction time (28). Yet, it was also found that rhythmic cueing facilitated redirecting attentional load to gait control, leading to a higher level of dynamic stability during walking (29). Although the use of rhythmic cueing could be considered an external agent for facilitating an automatized motor task, decreased gait speed even with the use of rhythmic cueing in older adults with Alzheimer’s disease indicates that the addition of rhythmic cueing may also increase attentional demands by involving cognitive processing of external auditory information in older populations with impaired executive control (30).

These findings support the potentials for expanding dual task methodologies from cognitive-motor dual task paradigms to those that utilize external resources, such as external rhythmic cueing. While walking with external auditory cueing has been repeatedly documented to be effective for gait control (27, 31), which enables its application to older adults with severe cognitive decline or increased fall risks, whether the effects are present in a dual task paradigm remain unclear. Therefore, this study aimed to examine how rhythm-motor tasks affect dual task performance and gait control, compared to a traditional cognitive-motor task. Also, it aimed to examine whether rhythm-motor tasks are correlated with traditional cognitive-motor task performance and cognitive measures. The findings of this study can be used to determine whether rhythm-motor tasks can be applied to the dual task paradigm for more diversified populations who have varying levels of cognitive and motor function or impairment. This study answered the following research questions:

1. Are there significant differences in DTC in walking speed or stride length among the participants in the rhythm-motor tasks and cognitive-motor tasks?
2. Are there significant differences in the ratio of stride length to walking speed as measured for walking stability among the participants in the rhythm-motor tasks and cognitive-motor tasks?
3. Are there significant correlations among the rhythm-motor tasks and cognitive-motor task?
4. Are there significant correlations among the DTC in walking parameters and cognitive and balance measures?

## Materials and Methods

### Participants

All procedures and ethical issues related to this study were reviewed and approved by the Institutional Review Board of Ewha Womans University (IRB No. 89-7). Community-dwelling healthy older adults aged 60 or over were recruited through flyers posted at senior apartments and senior community centers. Inclusion criteria were no previous diagnosis of dementia or neurological disorders and the ability to walk independently without aids. Participants were also screened for a Mini-Mental State Examination (MMSE) score over 24. In order to examine whether participants showed depressive symptoms, the Geriatric Depression Scale (GDS) was self-rated by participants (32). The GDS includes 30 items and asks individuals to rate whether they experienced a particular feeling over the past week. Participants were required to score less than 16 on the GDS. Initially, a total of 20 older adults with a mean age of 75.9 years (*SD* = 9.3) were recruited. One participant was excluded because of the MMSE score obtained was lower than 24. Another participant dropped out, since she did not want to complete the required tests and asked to leave the study. The remaining 18 participants were included in the final analysis.

### Measurement

#### Cognitive Assessment

For cognitive measures, the Digit Span Test (DST), Trail Making Test (TMT), and Wisconsin Card Sorting Test (WCST) were used. The DST (33, 34) assesses working memory and consists of two subtests: Digit Span Forward (DSF) and Digit Span Backward (DSB). The DSF tests the ability to recall a series of 3- to 9-digit numbers in a presented order, and the DSB tests the ability to recall a series of 2- to 8-digit numbers in a reverse order than presented.

The Korean version of the TMT was used in this study (35). Two subtests of the TMT (i.e., TMT-A and TMT-B) assess information processing speed, working memory, and executive function (36). The TMT-A tests the ability to draw a line connecting numbers sequentially from 1 to 15, and the TMT-B tests the ability to connect numbers and words for the days of the week alternatively (e.g., 1, Monday, 2, Tuesday, and so on in a sequential order). While both subtests indicate processing speed and cognitive flexibility by measuring the time to complete the tests, the TMT-B also indicates attentional control and set-shifting task performance (37).

Furthermore, the WCST (38, 39) tests the ability to match response cards to stimulus cards based on the sorting principle (e.g., colors, forms, or numbers of figures). Respondents are required to determine the initial sorting principle or newly changed principle based on feedback from the tester about whether each response is correct. The WCST measures executive functioning, including the ability to develop a problem-solving strategy, and preservative errors during the test indicate inhibitory attentional control and cognitive flexibility in set-shifting tasks.

#### Balance

To measure balance, the Timed Up and Go (TUG) test was implemented. TUG is a test that assesses balance (40) by measuring the time to complete the task of standing up from an arm-chair, walking a 3-m distance, turning back, and sitting back down. The measure is considered an indication of functional mobility and falls in everyday life (41). For older adults, a completion time of more than 13.5 s indicates a high risk of falling.

### Dual Task Measures

The single task was to walk along a 6-m walkway. Two types of concurrent tasks were used in this study: rhythm-motor tasks and a cognitive-motor task. For the rhythm-motor tasks, continuously matching to rhythmic cueing (Walk_RC_), striking two cylinder-type instruments together using both hands (Walk_IP_), and striking two instruments together while matching to the presented rhythmic cueing (Walk_IP+RC_) were presented. During these rhythm-motor tasks, rhythmic cueing was provided by a metronome. The tempo of rhythmic cueing was set to each participant’s preferred speed by measuring the cadence (steps per minute) when he or her was instructed to walk at his or her comfortable speed. A common cognitive-motor task found in the literature was used: counting forward from a two-digit number by 3s (Walk_Count.f3_).

Three types of measures were collected across the task conditions: gait parameters, DTC, and ratio of walking speed to stride length. For gait parameters, walking speed (m/s) and stride length (m) measured during two walking trials were averaged. In order to measure how much the addition of tasks affected gait, compared to baseline gait parameters that a participant performed without concurrent tasks, DTC was calculated for gait parameters (i.e., walking speed and stride length) in each dual task condition. DTC was the measure obtained by calculating the differences in gait parameter between single and dual task conditions using the following formula: (single − dual)/single*100. Finally, the ratio of stride length to walking speed was measured by calculating the stride length divided by walking speed. Given that changes in single gait parameter do not sufficiently reflect the gait pattern of an individual, such a measure of ratio (i.e., index for dynamic relationship between gait parameters) is considered indicative of the pattern of movement and postural control. Decreases in the measure of ratio may be indicative of decreased stride length and increased cautiousness while walking (8).

### Procedures

The current study was individually conducted in a quiet and spacious room within a participant’s apartment (12 participants) and at senior centers (six participants) where participants were recruited. In order to minimize the noise in the environment to an equivalent level across settings, isolated places without adjacent rooms where noise could be produced were selected. Also, in order to control the walking environment to a similar level across the participants, each room was examined to include a 6-m walkway without requiring participants to turn in another direction while walking. Written informed consent was obtained from each participant prior to participation in this study. After the consent process, demographic information was collected including sex, years of formal education, fall history, and medical history. Then measures of cognition, mobility and balance, and affect were implemented. During a single task condition, participants were instructed to walk at their comfortable speed along a 6-m walkway. Then, they were instructed to perform the additional task simultaneously and no instruction for prioritization of one task over the other (walking versus concurrent task) was provided. The order of presenting tasks was randomly determined for each participant prior to the test. In order to prevent participants’ fatigue from affecting gait parameters, there were 30-s breaks between the tasks and additional breaks while sitting were also considered if a participant requested a break or if he or she showed signs of shortness of breath or gait disturbances.

### Data Analysis

For measures of cognition and balance, descriptive data (i.e., mean and SD) were collected. For dual task-related parameters (i.e., DTC and ratio of stride length to walking speed), a repeated measures ANOVA was conducted to compare such measured values across the test conditions with the type of task as a within-group factor. Furthermore, correlations of DTC in each dual task condition with cognitive and balance measures were analyzed. All statistical tests were conducted using SPSS statistics 23 (IBM Corp., Armonk, NY, USA).

## Results

A total of 18 older adults were included in the final analysis. Demographic information of participants is displayed in Table 1. Their mean age was 75.3 years, and they had an average of 13.4 years of formal education. Among the participants, only three reported that they had fallen within the past 6 months. In this study, three cognitive measures (DST, TMT, and WCST) were administered. In terms of balance, the TUG test was used. The results of these measures are displayed in Table 2. The results of cognitive measures showed that participants were not cognitively deficient. In terms of the balance measure, given that individuals who complete the TUG test in 12 s or more are at risk for falling, the average time of 11.4 s for the participants to complete the test was indicative of a minimal risk for falling. When each participant was instructed to walk a 6-m distance at their preferred tempo, the average walking speed was 0.88 m/s (SD = 0.24), and their average stride length was 0.99 m (SD = 0.27). A Shapiro–Wilk test of normality was conducted to see if the measured gait parameters during self-paced walking were normally distributed so that the use of ANOVA tests was reasonable. The results of normality test showed that the data came from a normal distribution of gait parameters (*p* = 0.682 for walking speed and; *p* = 0.533 for step length).

**Table 1:** Demographic information of participants.

Parameter	*N* = 18
Sex, M:F	3:15
Age, years (M ± SD)	75.3 ± 9.2
Education, years (M ± SD)	13.4 ± 3.6
Number of falls in past 6 months, *n* (%)
0	15 (83.3%)
1	2 (11.1%)
2	1 (5.6%)
MMSE (*M* ± *SD*)	28.1 ± 1.3
GDS (*M* ± *SD*)	6.4 ± 6.8

*MMSE, mini-mental state examination; GDS, geriatric depression scale*.

**Table 2:** Cognitive, affect, and balance measures of participants.

Parameter	*N* = 18
**Cognitive**	
DSF, number of digits recalled	6.4 ± 1.4
DSB, number of digits recalled	4.3 ± 1.2
TMT-A, seconds	23.9 ± 8.4
TMT-B, seconds	65.0 ± 74.2
WCST-correct response	37.7 ± 11.7
WCST-errors	26.3 ± 11.7
WCST-preservative errors	21.6 ± 17.6
**Balance**	
TUG	11.4 ± 0.5

*DSF, digit span forward; DSB, digit span backward; TMT, trail making test; WCST, Wisconsin card sorting test; TUG, timed up and go*.

### DTC in Gait Parameters

**Figure 1:**
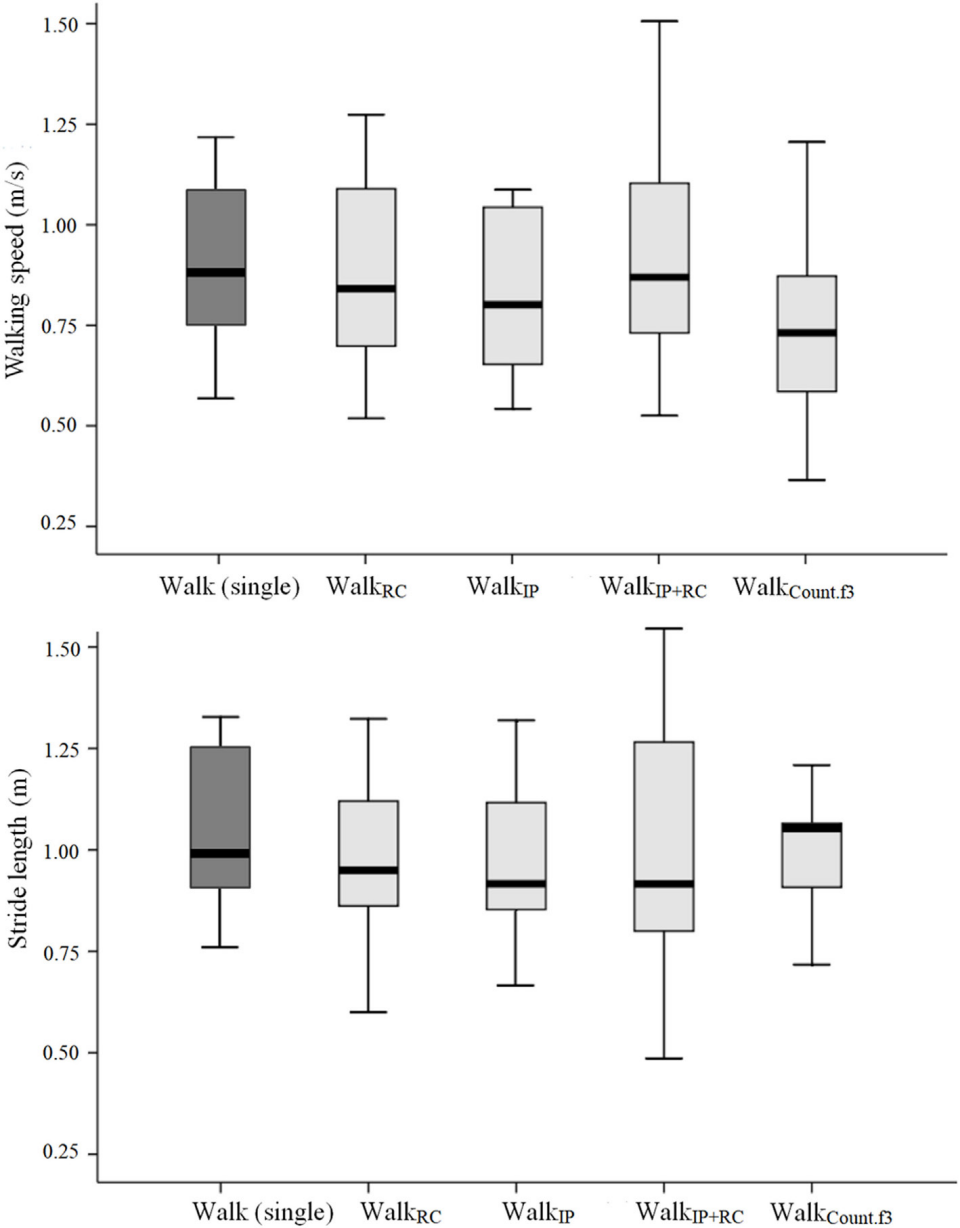
Gait parameters measured during each task condition. Top panel presents the walking speed and bottom panel presents the stride length.

For each task condition, the measured walking speed and stride length are displayed in Table 3. The highest walking speed was observed during self-paced walking as a single task and the lowest walking speed was measured during the Walk_Count.f3_ task. For stride length, participants were found to show the longest stride length during self-paced walking and the shortest stride length during the Walk_IP+RC_ task (see Figure 1).

**Table 3:** Gait parameters during single and dual tasks.

Task	Walking speed (m/s) M ± SD	Stride length (m) M ± SD
Walk (single)	0.88 ± 0.24	0.99 ± 0.27
Walk_RC_	0.86 ± 0.25	0.94 ± 0.24
Walk_IP_	0.81 ± 0.21	0.91 ± 0.24
Walk_IP+RC_	0.83 ± 0.22	0.89 ± 0.25
Walk_Count.f3_	0.69 ± 0.20	0.97 ± 0.26

*RC, rhythmic cueing; IP, instrument playing; Count f3, counting forward by 3s*.

Given DTC measures how the addition of concurrent task while walking affects the required demands of attending to gait and whether such demand interferes with gait performance, DTC in walking speed was analyzed across the task conditions. The least interference (e.g., the least cost) was observed with the Walk_RC_ condition, which indicates that the difference from the single walking task was the least. The greatest cost was observed with the Walk_Count.f3_ condition: the degree of decreased walking speed was the greatest in this condition, compared to the single walking task, indicating that the attentional interference of gait was the greatest (see Table 4). In terms of stride length, the greatest DTC was observed with the Walk_IP+RC_ condition followed by the Walk_RC_ condition, with increased DTC indicating decreased stride length.

**Table 4:** Dual task costs depending on the dual task type.

Task	Walking speed (m/s) M ± SD	Stride length (m) M ± SD
Walk_RC_	2.19 ± 12.91	4.21 ± 11.65
Walk_IP_	6.86 ± 8.26	7.09 ± 7.10
Walk_IP+RC_	5.68 ± 11.17	9.28 ± 12.20
Walk_Count.f3_	20.30 ± 15.76	1.33 ± 9.73

*RC, rhythmic cueing; IP, instrument playing; Count f3, counting forward by 3s*.

The results of a repeated measures ANOVA showed that in terms of walking speed, there was a significant main effect for task, *F*(3, 51) = 9.400, *p* < 0.001, eta^2^ = 0.356. A *post hoc* analysis with Bonferroni’s correction demonstrated that DTC in walking speed during the Walk_Count.f3_ tasks was significantly slower than during other dual tasks (*p* = 0.006 for comparison between Walk_RC_ and Walk_Count.f3_; *p* = 0.033 for comparison between Walk_IP_ and Walk_Count.f3_; *p* = 0.013 for comparison between Walk_IP+RC_ and Walk_Count.f3_). For stride length, the main effect of the task was also statistically significant, *F*(3, 51) = 3.989, *p* = 0.021, eta^2^ = 0.190. A *post hoc* analysis demonstrated that DTC in stride length during the Walk_Count.f3_ tasks was significantly shorter than during Walk_IP_ (*p* = 0.036).

**Figure 2:**
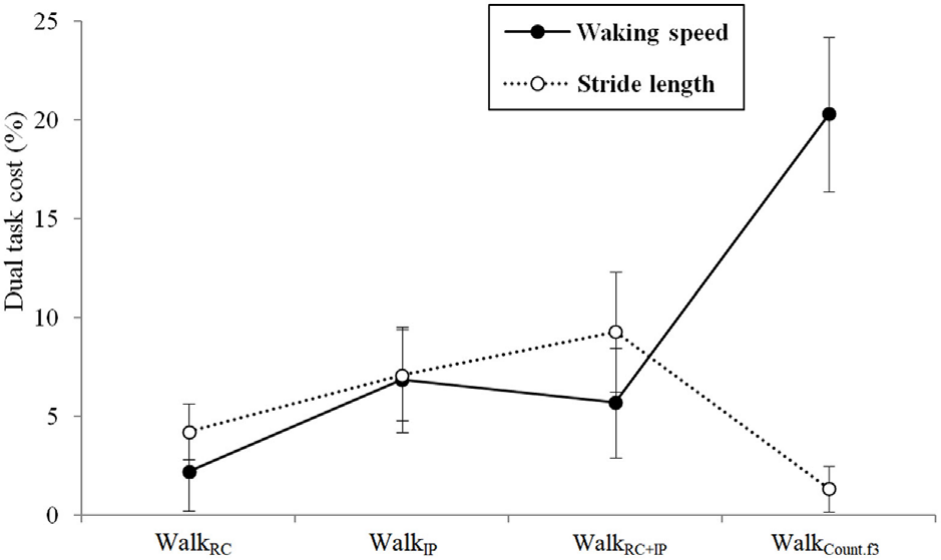
Dual task costs in walking speed and stride length depending on the task.

The results also showed that depending on the task, the difference in DTC between walking speed and stride length varied (see Figure 2). For the single walking task, DTC was the least for both walking speed and stride length. For the Walk_Count.f3_ tasks, while DTC for walking speed increased greatly, the degree of increase was the least for stride length.

### Ratio of Step Length to Walking Speed during Single and Dual Tasks

**Figure 3:**
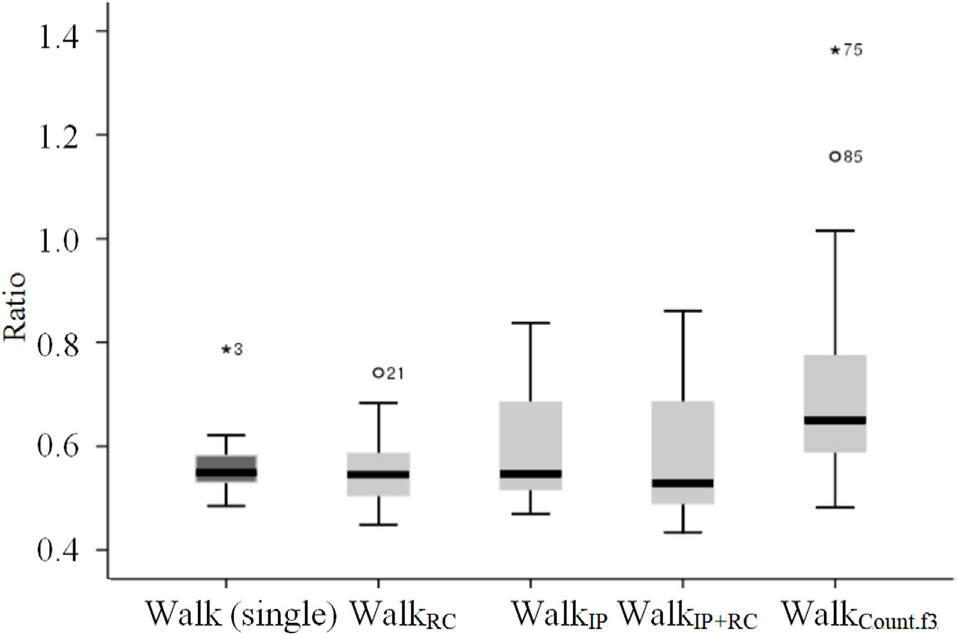
Ratio of stride length to walking speed depending on the task.

Given the differences in DTC between walking speed and stride length, the ratio of stride length to walking speed was analyzed to see whether the task type influenced this measure. The ratios of stride length to walking speed are displayed in Table 5 and Figure 3. The ratio of stride length to walking speed was the greatest in the Walk_Count.f3_ condition. For the percent change to the single walking task, it was 99.1% during the Walk_RC_ condition and increased to 133.7% during the Walk_Count.f3_ condition.

**Table 5:** Ratio of stride length to walking speed during single and dual tasks.

Parameter	Stride length/walking speed M ± SD	Percentage change to single task (%) M ± SD
Walk (single)	0.57 ± 0.06	–
Walk_RC_	0.56 ± 0.07	99.1 ± 6.7
Walk_IP_	0.60 ± 0.11	106.1 ± 18.4
Walk_IP+RC_	0.59 ± 0.12	103.6 ± 19.0
Walk_Count.f3_	0.76 ± 0.25	133.7 ± 34.7

*RC, rhythmic cueing; IP, instrument playing; Count f3, counting forward by 3s*.

The results of repeated measures ANOVA showed that there was a significant main effect for task, *F*(4, 68) = 10.220, *p* < 0.001, eta^2^ = 0.375. A *post hoc* analysis with Bonferroni’s correction showed that the Walk_Count.f3_ condition elicited a significantly greater ratio than the single walking task (*p* = 0.013) and other dual tasks (*p* = 0.007 for comparison between Walk_RC_ and Walk_Count.f3_; *p* = 0.026 for comparison between Walk_IP+RC_ and Walk_Count.f3_) except for the Walk_IP_ condition.

### Correlation among Rhythm-Motor and Cognitive-Motor Dual Tasks

Pearson’s correlation results are displayed in Table 6. In terms of DTC for walking speed, Walk_RC_ was found to be moderately correlated with Walk_IP+RC_. Other pairs did not reach statistical significance. For the ratio measure, all pairs showed significant correlations. This indicates that the measures for gait control during rhythm-motor tasks and cognitive-motor tasks are intercorrelated and different rhythm-motor tasks are also intercorrelated.

**Table 6:** Correlation among rhythm-motor and cognitive-motor dual tasks.

	Walk_IP_ *r* (*p*)	Walk_IP+RC_ *r* (*p*)	Walk_Count.f3_ *r* (*p*)
**Dual task costs in walking speed**			
Walk_RC_	0.088 (0.728)	0.604** (0.008)	0.104 (0.681)
Walk_IP_	–	0.270 (0.279)	−0.029 (0.910)
Walk_IP+RC_	–	–	0.289 (0.246)
**Ratio of stride length to walking speed**			
Walk_RC_	0.469* (0.049)	0.547* (0.019)	0.753*** (<0.001)
Walk_IP_	–	0.966*** (<0.001)	0.549* (0.018)
Walk_IP+RC_	–	–	0.585* (0.011)

*p < 0.05; **p < 0.01; ***p < 0.001.

### Correlation of DTC with Cognitive and Balance Measures

Pearson’s correlation results are displayed in Table 7. DTC for walking speed during Walk_IP_ was found to be negatively correlated with DSB. This indicates that as the level of interference due to dual task performance decreases during the Walk_IP_ condition, the number of recalled numbers backwards increases. For DTC during the Walk_IP+RC_ condition, moderate correlations were found with TMT-A and TMT-B, indicating that as DTC increases, the time to complete the task of connecting numbers (or numbers and letters) sequentially also increased. DTC for Walk_IP+RC_ was found to be correlated with the number of digits recalled (DSF).

**Table 7:** Correlation between dual task costs (DTC) in walking speed and cognitive measures.

DTC	Cognitive measure							
	Timed up and go *r* (*p*)	Digit span forward *r* (*p*)	Digit span backward *r* (*p*)	Trail making test (TMT)-A *r* (*p*)	TMT-B *r* (*p*)	Wisconsin card sorting test (WCST)-correct *r* (*p*)	WCST-error *r* (*p*)	WCST-p.error *r* (*p*)
Walk_RC_	0.186 (0.725)	−0.047 (0.854)	−0.037 (0.885)	0.381 (0.119)	0.045 (0.859)	−0.171 (0.496)	0.171 (0.496)	0.255 (0.322)
Walk_IP_	0.052 (0.922)	−0.289 (0.244)	−0.676** (0.002)	0.387 (0.113)	0.249 (0.320)	−0.262 (0.293)	0.262 (0.203)	0.266 (0.302)
Walk_IP+RC_	0.122 (0.817)	−0.070 (0.784)	0.045 (0.861)	0.697** (0.001)	0.456^†^ (0.057)	−0.129 (0.610)	0.129 (0.610)	0.208 (0.422)
Walk_Count.f3_	0.168 (0.751)	−0.473* (0.047)	0.042 (0.870)	0.119 (0.640)	0.025 (0.922)	0.154 (0.541)	−0.154 (0.541)	−0.067 (0.798)

*p < 0.05; **p < 0.01; ^†^p < 0.10.

*Shaded values indicate the case with correlations significant at p = .05*.

## Discussion

### DTC in Gait Parameters

The results of this study showed that participants tended to walk more slowly and with shorter steps during dual task conditions. Also, when comparing DTCs across task conditions, it was found that the task type significantly affected this measure. In terms of walking speed, the measure of DTC was significantly greater during Walk_Count.f3_ (i.e., slower walking) than during the rhythm-motor tasks. In terms of stride length, DTC was the greatest in the Walk_IP_ condition (i.e., decreased stride length), leading to a significant difference between this and the Walk_Count.f3_ condition, which elicited the least DTC. Given that the measure of DTC represents the level of interference or attentional resources that a concurrent task recruits, the results indicate that depending on the type of concurrent task, attentional control abilities are required at different levels. A previous meta-analysis demonstrated that when a concurrent cognitive task involves internal information processing (e.g., such processing to be manipulated while holding the information), the level of interference with gait control increases, compared to when a secondary task involves response to an external stimulus (24). In the meta-analysis, dual task interference increased when mental tracking or a verbal fluency task was presented, compared to when a reaction time task was presented.

In this study, the task of counting numbers forward by 3s required mental tracking that involved holding information internally and manipulating the information (42). Meanwhile, tapping instruments together while walking involved interlimb coordination and matching to rhythmic cueing involved a response to external stimuli. Such differences in information processing may differentially affect dual task interference. In addition, when looking at rhythm-motor tasks, DTC in walking speed increased during Walk_IP_ and Walk_IP+RC_, compared to during Walk_RC_. These results indicate that interlimb coordination involved increased attentional control, compared to when responding to external auditory cueing. The task of interlimb coordination plus matching to rhythmic cueing requires a similar level of attentional control to interlimb coordination without matching to rhythmic cueing, which indicates that the addition of rhythmic cueing may not significantly affect the recruitment of additional attentional resources.

For stride length, the tendency of changes in DTC across the tasks was not similar to the results in walking speed. The Walk_Count.f3_ condition, which elicited the greatest DTC in walking speed, did not lead to increases in DTC. Rather, the Walk_IP_ condition elicited the greatest DTC. While participants tended to greatly slow down their walking during the Walk_Count.f3_ task, they tended to decrease their stride length during the rhythm-motor tasks. Such results indicate that during the mental task of the Walk_Count.f3_ condition, participants tended to prioritize cognitive tasks by sacrificing their walking speed; they tended to prioritize motor tasks during Walk_IP_ by modifying their gait patterns leading to safer gait.

### Ratio of Step Length to Walking Speed during Single and Dual Tasks

While the amount of changes in gait parameters in dual task conditions indicates the degree of cognitive-motor interference, changing direction in each parameter and the combination of such changes indicate the level of gait stability to be affected. Thus, in this study, the ratio of step length to walking speed was analyzed across the task conditions. The Walk_Count.f3_ condition elicited increased ratio when compared to the single walking task and rhythm-motor dual tasks except Walk_IP_. When examining the differences among the rhythm-motor dual tasks, greater increase in the ratio measure was observed during the Walk_IP_ condition, compared to the Walk_RC_ and Walk_IP+RC_ conditions. These increases in ratio were attributed to decrease in walking speed in Walk_Count.f3_ condition, while changes in rhythm-motor task conditions were attributed to decrease in stride length. In this study, participants tended to maintain stride length while greatly slowing down their walking during the Walk_Count.f3_ task. Furthermore, during rhythm-motor tasks, participants tended to maintain walking speed while decreasing their stride length.

Previous research implied that adaptive gait in response to perturbations involves shorter stride length and increased gait frequency while keeping the walking speed constant (9). Accordingly, while decrease in walking speed may be indicative of increased interference, decrease in stride length may be indicative of cautious walking as an adaptive strategy. In this study, the concurrent task of counting contributed to increased interference with adaptive gait control as well as involvement of increased attentional resources. Decreased ratio during the Walk_RC_ (compared to single walking) and Walk_IP+RC_ (compared to Walk_IP_) due to changes in stride length without changes in walking speed could be explained as attempts to ensure safe gait when additional cognitive resources are involved. On the contrary, the Walk_IP_ and Walk_Count.f3_ tasks elicited increased ratio compared to single walking. In the case of Walk_IP_, although decreased stride length was observed as an adaptation for gait stability, decreases in walking speed were also observed, which led to interference with effective gait control [also found in Ref. (43)], compared to other rhythm-motor tasks. These results support previous studies demonstrating cued walking as effective for gait control (28, 44). This also gives rise to the potentials for rhythm-motor tasks as a dual task for facilitating adjustment of gait in response to increased attentional demands. Further studies are needed to investigate such direct effects of rhythm-motor tasks and whether they can be applied to individuals with neurological disorders using a dual task paradigm.

### Correlation of Rhythm-Motor Task-Related Measures with Other Parameters

The ratio measure during all rhythm-motor tasks was found to be significantly correlated with the Walk_Count.f3_ task. Given that Walk_Count.f3_ is one of the most commonly used tasks in the literature, this result indicates that gait control during walking with rhythm-motor tasks may be associated with such control during cognitive-motor dual tasks, supporting the potential for using rhythm-motor tasks as a dual task.

The results of the correlation analysis between DTC with cognitive and balance measures showed that DTC in walking speed during Walk_IP_ was found to be negatively correlated with DSB. For DTC during the Walk_IP+RC_ condition, significant correlations were found with TMT-A and TMT-B. Given that the DSB and TMT tests require inhibitory control and cognitive flexibility, the observed significant correlations with Walk_IP_ and Walk_IP+RC_ support that rhythm-motor tasks may involve dynamic control of attention (45) similar to cognitive measures by coordinating interlimbs in response to external information. Other research reported that the transfer of executive control skills to the gross motor domain (46), which supports intervening in executive control, would contribute to increased gait control and decreased fall risks.

The results of this study imply that rhythm-motor dual tasks may be effectively incorporated into the dual task paradigm that targets fall prevention for older populations with varying needs for cognitive and motor control. Despite their potential, this should be applied with caution. Although Walk_Count.f3_ is one of the most commonly used tasks as a mental tracking task, this study did not compare the potential of rhythm-motor task for cognitive and motor control with all other types of traditional cognitive-motor tasks (e.g., verbal fluency task), which may be different from Walk_Count.f3_ in terms of the level of complexity or the amount of cognitive load involved. Also, the addition of rhythmic cueing to a traditional cognitive-motor task would present more useful information on how a rhythm-motor task can mediate dual task performance at different levels.

## Conclusion

This study demonstrated that rhythm-motor tasks involve cognitive control of attentional resources supporting their application to traditional cognitive-motor tasks for dual task intervention. Given that the inclusion of dual tasks, not intervention in single cognitive or motor functioning, leads to improvement in dual task performance and fall prevention, the results with rhythm-motor tasks support their applicability to intervening in such needs of older populations. Particularly, since rhythm-motor tasks have the potential to intervene in maintaining gait stability in a dual task paradigm, its applicability could also be supported in older populations with varying needs for cognitive and/or motor control, including older populations with cognitive impairment or neurological disorders. In addition, the results of this study present the implication for utilizing rhythm-motor tasks at different levels and in combination with other cognitive-motor tasks. Despite the promising findings in this study, the results should be generalized with caution. Increased sample size and inclusion of older adults with varying levels of cognitive aging in future studies will present how the use of rhythmic cueing as an external source could intervene in cognitive and gait control. While this study included rhythm-motor tasks as separate tasks, the combination of cognitive-motor dual tasks with the task to match to rhythmic cueing will clarify the potential of adding external auditory sources as a compensatory strategy to maintain gait stability within the dual task paradigm.

## Ethics Statement

All procedures and ethical issues related to this study were reviewed and approved by the Institutional Review Board of Ewha Womans University (IRB No. 89-7).

## Author Contributions

SJK, S-RC, and GEY contributed to study conception and design, data acquisition and analysis, and manuscript writing.

## Conflict of Interest Statement

The authors declare that the research was conducted in the absence of any commercial or financial relationships that could be construed as a potential conflict of interest.
